# Impacts of Random Atomic Defects on Critical Buckling Stress of Graphene under Different Boundary Conditions

**DOI:** 10.3390/nano13091499

**Published:** 2023-04-27

**Authors:** Jiajia Shi, Liu Chu, Zhengyu Yu, Eduardo Souza de Cursi

**Affiliations:** 1 School of Transportation and Civil Engineering, Nantong University, Nantong 226001, China; 2 Faculty of Engineering and Information Technology, University of Technology Sydney, Sydney, NSW 2050, Australia; 3 Département Mécanique, Institut National des Sciences Appliquées de Rouen, 76800 Rouen, France

**Keywords:** buckled graphene, random porosities, stochastic finite element model

## Abstract

Buckled graphene has potential applications in energy harvest, storage, conversion, and hydrogen storage. The investigation and quantification analysis of the random porosity in buckled graphene not only contributes to the performance reliability evaluation, but it also provides important references for artificial functionalization. This paper proposes a stochastic finite element model to quantify the randomly distributed porosities in pristine graphene. The Monte Carlo stochastic sampling process is combined with finite element computation to simulate the mechanical property of buckled graphene. Different boundary conditions are considered, and the corresponding results are compared. The impacts of random porosities on the buckling patterns are recorded and analyzed. Based on the large sampling space provided by the stochastic finite element model, the discrepancies caused by the number of random porosities are discussed. The possibility of strengthening effects in critical buckling stress is tracked in the large sampling space. The distinguishable interval ranges of probability density distribution for the relative variation of the critical buckling stress prove the promising potential of artificial control by the atomic vacancy amounts. In addition, the approximated Gaussian density distribution of critical buckling stress demonstrates the stochastic sampling efficiency by the Monte Carlo method and the artificial controllability of porous graphene. The results of this work provide new ideas for understanding the random porosities in buckled graphene and provide a basis for artificial functionalization through porosity controlling.

## 1. Introduction

Buckling is one of the most common phenomena in low-dimensional nanomaterials. Generally, buckling induces delamination [1], instability [2], and wrinkles [3] in graphene sheets and graphene-based composites. However, besides the negative effects, buckled graphene sheets have positive applications in energy harvest [4], hydrogen storage [5], and artificial functionalization design [6,7]. The wasted mechanical energy can be collected and stored in the form of internal potential and converted into concentrated kinetic or thermal energy through buckled graphene. In addition, the buckled graphene sheets are predicted to perform fast hydrogen storage and release by the corrugation formation and controlled inversion of curvature. Furthermore, the buckling deformation of graphene is also a potential and feasible way for artificial nanomaterial manufacturing and functionalization [8,9]. Therefore, the investigation of buckling in graphene is not only important for the instability, wrinkle, and corrugation mechanism, but it is also directly related to the potential applications in energy harvest, hydrogen storage, and artificial functionalization.

The causes of bucking in graphene vary [10]. Bending-induced local buckling is observed experimentally [11] and characterized by theoretical modeling and molecular dynamics simulation [12,13,14]. In addition, buckling of dislocation in graphene is also reported [15]. The shapes of the deformations of the buckled graphene are sensitive to external boundary conditions, grain boundaries [16], compressive tension, porosity, defects in the microstructure [17], etc. From the perspective of numerical simulation, the molecular mechanic method is performed based on the Morse potential functions with the parameter approximation for buckling analysis [18]. The analytical approach is also developed to analyze the stability of defective annular graphene sheets. The nonlocal thin plate theory is combined with the translational addition theorem, while the defects are modeled as eccentric holes [19]. In addition, the van der Waals forces of the bonded interaction between atoms are simplified as the truss or beam elements to study the impacts on buckling strength [20]. Therefore, buckling analysis is a challenging issue related to the instability, boundary conditions, and local microstructures (defects, porosity, dislocations, etc.), thereby deserving more concern.

For the porosities or the local atomic vacancy defects in graphene, the conventional and analytical deterministic models have a shortcoming in the random distribution quantification and computational cost [21]. As mentioned above, vacancy defects are supposed to be eccentric holes in analytical methods [16]. However, the shapes and sizes of eccentric holes are not appropriate to describe the micro geometrical characteristics of porosities and vacancy defects. A large number of possible lattice isomers of nanopore topological shape also make difficulties in the interpretation of experimental and simulated data [22]. On the other hand, neglecting the random distribution of porosity and simplifying the random locations into specific locations causes the corresponding results to be limited in the deterministic situation and unable to represent general situations. Furthermore, the interactions between the randomly distributed atomic vacancy defects and their comprehensive effects on the buckling stress of graphene are challenging issues, requiring feasible solutions [23]. For example, the defective graphene sheets exhibit a negative Poisson’s ratio with auxetic behavior [24]. Therefore, developing efficient methods to quantify and propagate the random porosities in the current common models is necessary.

Compared with molecular dynamics, the finite element model of graphene has competitive merits in computational costs of buckling analysis, especially for the tremendous stochastic sampling procedures [25]. Moreover, the finite element model is more convenient for random factor quantification and propagation than the analytical models. Different types of porosity distributions and graphene platelet dispersion patterns are feasible to be introduced and implemented in the numerical simulation by the finite element method [26,27,28]. The nonlinear, irregular, and random geometrical complexity are settled for further computation based on the finite element model. Based on the continuum theory [29], the Monte Carlo stochastic sampling process is performed to propagate the random atomic vacancy defects in the finite element model of pristine graphene.

In this paper, a stochastic finite element model is proposed for the efficient quantification and propagation of the random porosities in buckled graphene. The main contents include the graphene geometrical characterization, method description, and result discussion. The buckling patterns of porous graphene are provided and compared under different boundary conditions. Based on the huge sampling space provided by the stochastic finite element model, the discrepancies caused by the numbers of random porosities are discussed. The work provides an important reference for the exploration of buckled graphene in energy harvest, hydrogen storage, and artificial functionalization.

## 2. Graphene Geometrical Configuration

The carbon atoms in graphene are combined with the solid covalent bonds to form the periodic honeycomb lattice, which results in extraordinary material and mechanical properties. In order to describe the special periodic microstructure, the beam element is used to simplify the carbon covalent bonds in the finite element model. The corresponding material and geometrical parameters are settled according to the specific values reported in the literature [30,31].

In addition, the percentage of random porosities (*P*), $P=\frac{N_{v}}{N_{a}}$, is gradually increased to observe the impact discrepancies. The number of carbon atoms in pristine graphene (*N_a_*) is a deterministic value. The number of the vacancy defect atoms $N_{v}$ is calculated by the multiplication of the *P* and *N_a_*.

**Figure 1:**
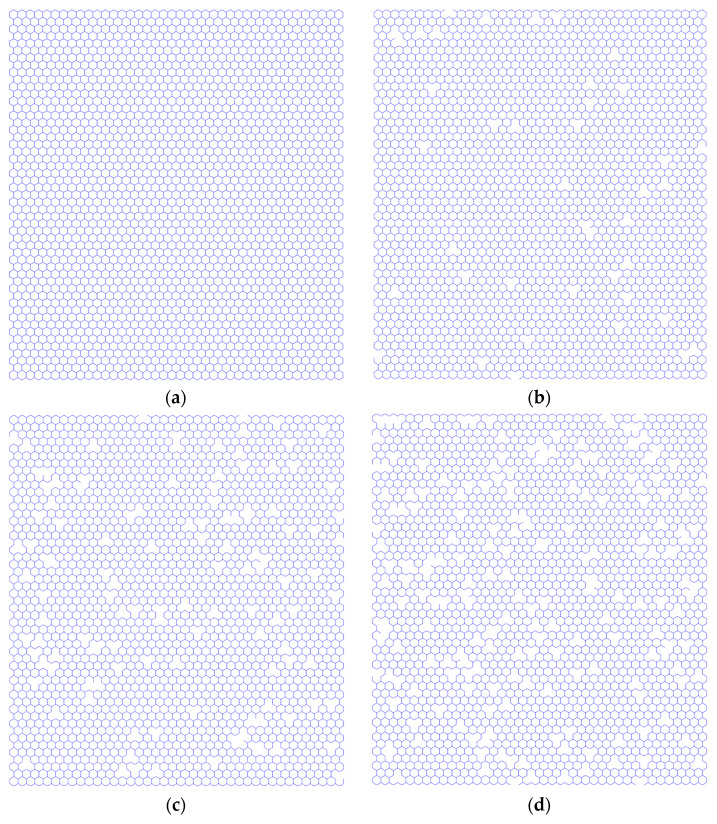
Graphene geometrical configurations ((**a**) is for the pristine graphene, and (**b**–**d**) are for defective graphene with 1%, 3%, and 5% atomic vacancy defects, respectively).

Each carbon atom in the graphene lattice is numbered by the series. The random numbers are generated by the Monte Carlo stochastic sampling procedure according to the value of $N_{v}$. Then, the random numbers are mapped to the graphene lattice, and the corresponding carbon atoms are marked and selected as atomic vacancy defects. Three related covalent carbon bonds are interrupted because of the disappearance of the carbon atoms as vacancy defects. Since the random numbers are stochastically selected by the Monte Carlo sampling method, the atomic vacancy defects are distributed randomly in the graphene lattice. Furthermore, the more complicated porosities can be generated based on the Monte Carlo sampling. When the atomic vacancy defects are adjacent, the porosities in graphene are formed in different sizes and shapes. The numerous repetitions of random propagation of atomic vacancy defects in the pristine graphene based on the Monte Carlo simulation are performed to make sure the specific possibilities are included in the sample space. As shown in Figure 1, the characteristic periodic honeycomb lattice of pristine graphene, as well as porous graphene with different amounts of random atomic vacancies, are presented.

## 3. Boundary Conditions

**Figure 2:**
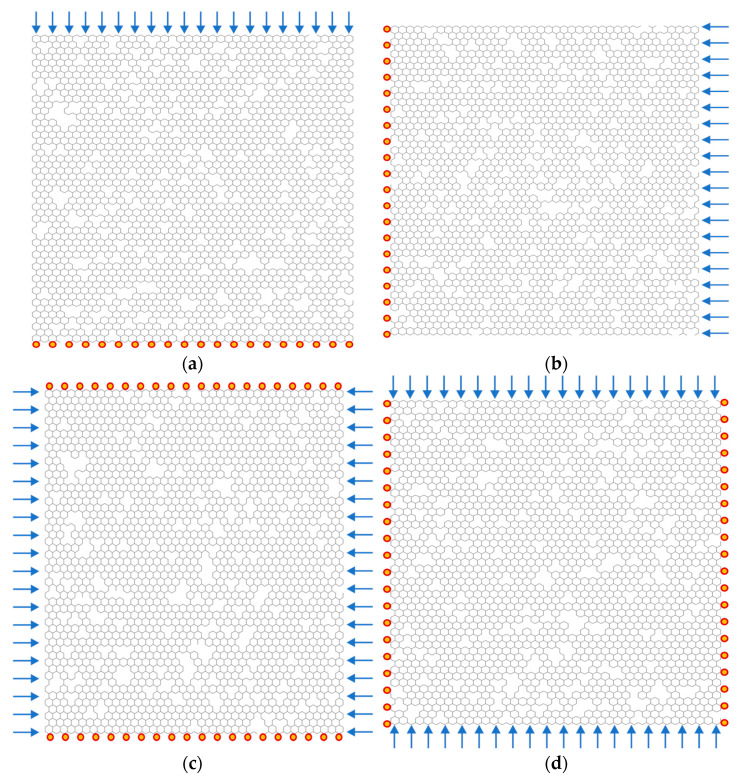
The schematic of porous graphene with different boundary conditions ((**a**–**d**) represent B1, B2, B3, and B4, respectively).

In order to compare and analyze the behaviors of porous graphene under diverse operating environments, four different boundary conditions are taken into consideration. In the first case, the boundary condition (B1) is settled as in Figure 2a, all the nodes in the lower edge of the porous graphene are clamped, and the six degrees of freedom, including the displacement and rotation in the x, y, z directions, are constraints. On the upper edge of the porous graphene, the uniformly distributed external forces F are loaded. The direction of external forces F is presented in Figure 2, as compressive uniform load, while the modulus of external forces F is supposed to be unit one. On the contrary, the boundary condition (B2) in the second case is settled on the left and right edges with clamped constraints and external forces, respectively. Since the deformation shapes of the buckled graphene nanoribbons depends on the boundary conditions [16], the in-plane compressive forces F are performed in the porous graphene, as shown in Figure 2.

In addition, the boundary condition (B3) in the third case is more complicated than that in B1 and B2. As presented in Figure 2c, all the nodes in the lower and upper edges of porous graphene are clamped, where the displacements and rotations are constraints. The external forces F are loaded in both the left and right edges. In the fourth case, the boundary condition (B4) is similar to that in B3. However, the constraints are settled in the left and edges, and the external forces F are loaded in the upper and lower edges of porous graphene. From the perspective of boundary conditions, the operating environments of porous graphene in B1 and B2 are supposed to be one group, while B3 and B4 are concluded as another comparable group.

**Figure 3:**
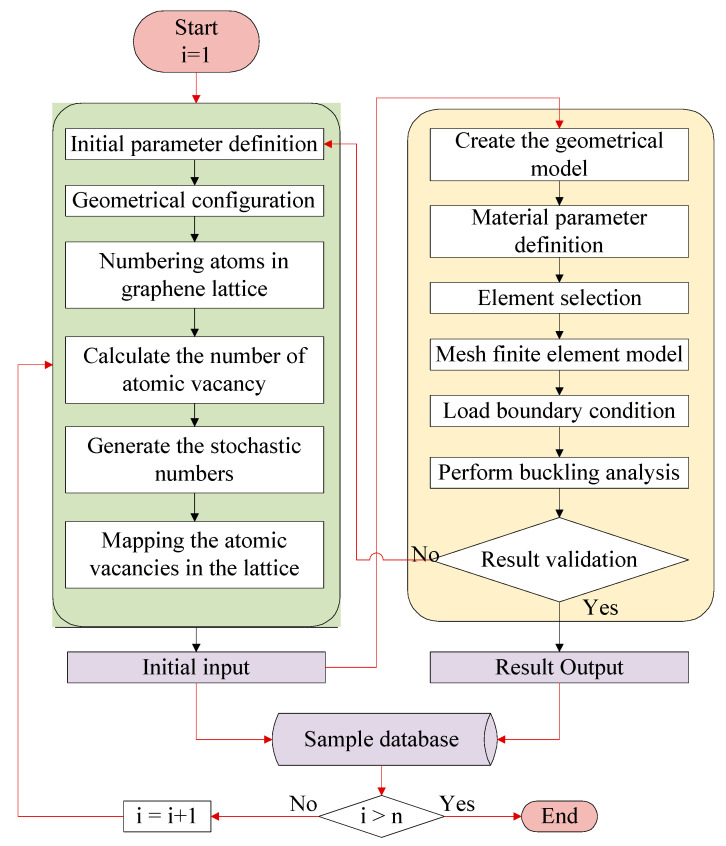
The flowchart of the stochastic finite element model for buckling analysis of porous graphene.

Furthermore, the implementation program and numerical computation are performed by the integration of Matlab (Version 2020a) and ANSYS (Version 14.5) commercial software. The corresponding parameter definition, parameter value assignment, and Monte Carlo stochastic sampling are completed under the Matlab programming environment. ANSYS parameter design language (APDL) is used to create the finite element model for porous graphene and perform mechanical computation for elastic buckling. The flowchart of the stochastic finite element model by the combination of Monte Carlo stochastic sampling with the finite element computation is shown in Figure 3. BEAM188 element is selected to mesh the carbon-carbon bonds in graphene with six degrees of freedom. There is a total of 6226 beam elements with 16,664 nodes in the model of pristine graphene. As the porosity amount increases in graphene, the number of beam elements and nodes decreases with discrepancy.

In Figure 3, the programming procedures in the green box are performed in the Matlab environment, while the progress in the yellow box is implemented by the ANSYS parameter design language. The interactive integration between Matlab and ANSYS is required both in the result validation and sample database confirmation. The critical buckling stress computation in the finite element model of graphene is based on the Lanczos algorithm [31,32]. The effective buckling length $L_{e}$ measures the node-to-node distance of the buckling mode and refers to the denominator in the Euler buckling formula $P_{cr}=\frac{\pi^{2}EI}{(KL{)}^{2}}\begin{array}{cc} , & L_{e}=KL \end{array}$, where K is the symmetric positive semidefinite stiffness matrix, L is the length of the beam, E is the elastic modulus, and I is the moment of inertia. Based on the finite element computation, the critical buckling stress, the displacements, and the rotation contours of porous graphene are provided.

The critical buckling stress is the key factor in controlling the elastic recycling process efficiently [33]. The eigenvalue buckling is elastic and recyclable, which is an important mechanism for energy and hydrogen storage and conversion recycling. In order to compare the impacts of the boundary condition and the atomic vacancies, the relative value and relative variation for critical buckling stress are introduced as the non-dimensional parameters, $R=\frac{S_{d}}{S_{p}}\begin{array}{cc} , & \delta =\frac{S_{d}-S_{p}}{S_{p}} \end{array}$, which are computed by the ratio between the results of porous graphene $S_{d}$ and that of pristine graphene $S_{p}$ under the same boundary condition.

## 4. Results and Discussion

### 4.1. Buckling Patterns

**Figure 4:**
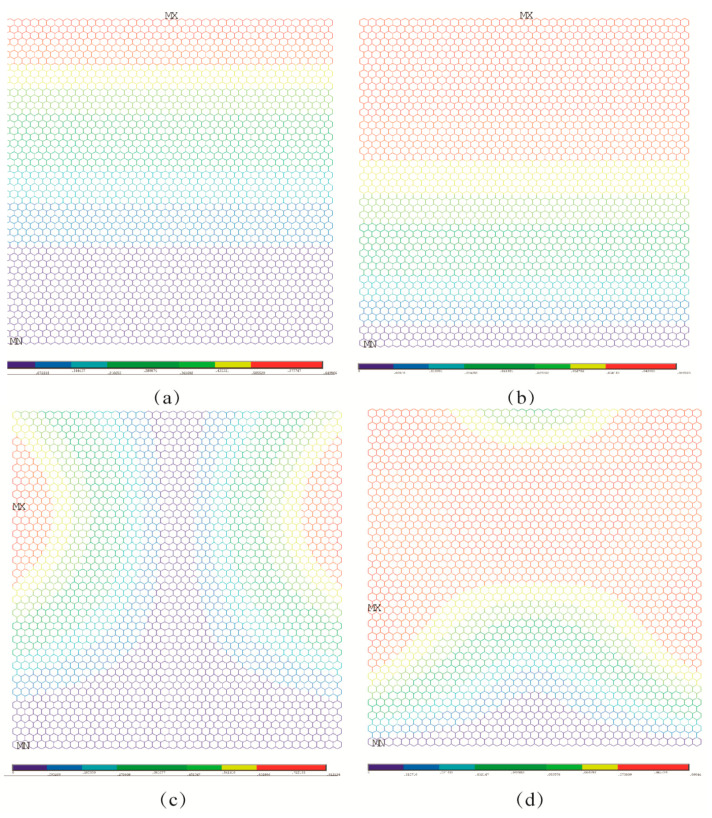

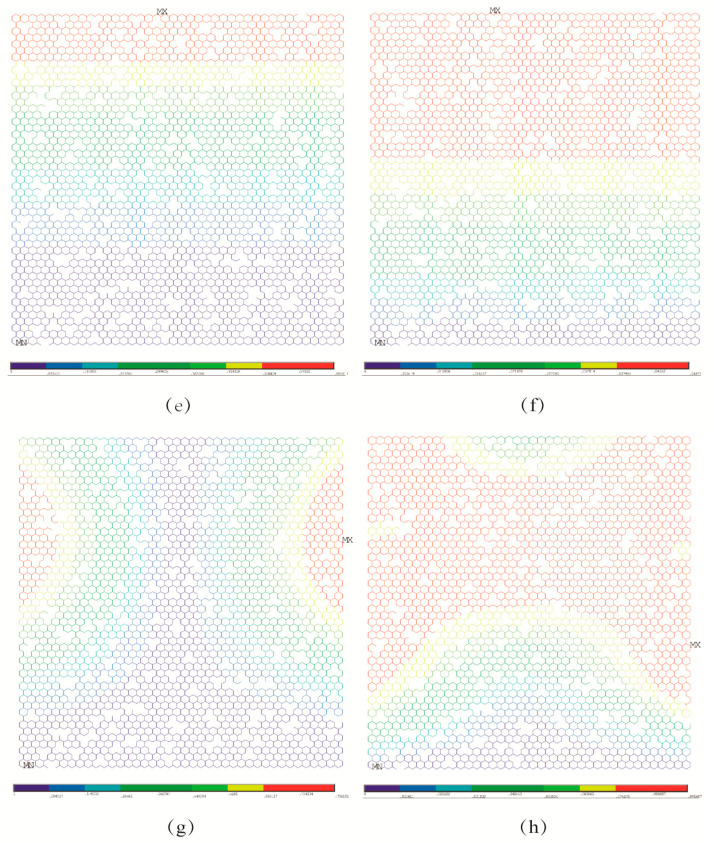
Comparison for buckling patterns under the first boundary condition ((**a**–**d**) represent the pristine graphene, while (**e**–**h**) represent the porous graphene with 5% atomic vacancy defects, respectively).

**Figure 5:**
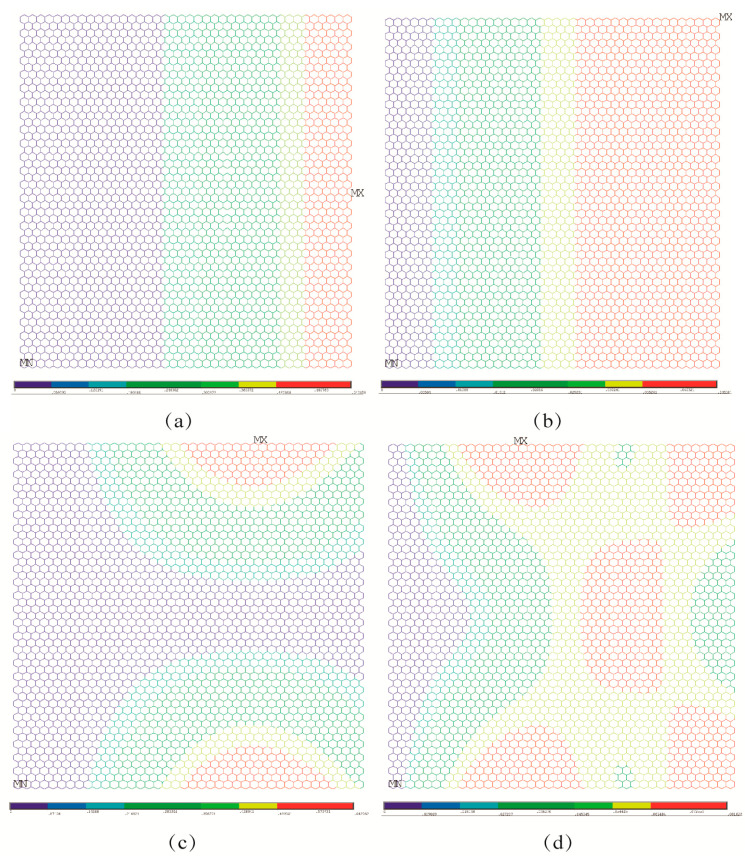

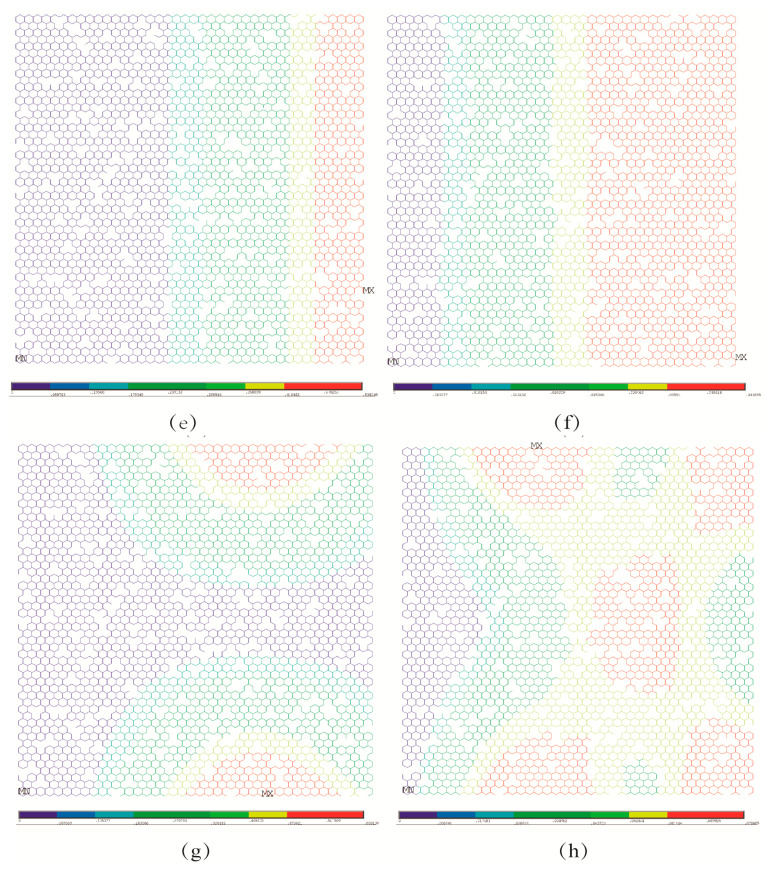
Comparison for buckling patterns under the second boundary condition ((**a**–**d**) represent the pristine graphene, while (**e**–**h**) represent the porous graphene with 5% atomic vacancy defects, respectively).

**Figure 6:**
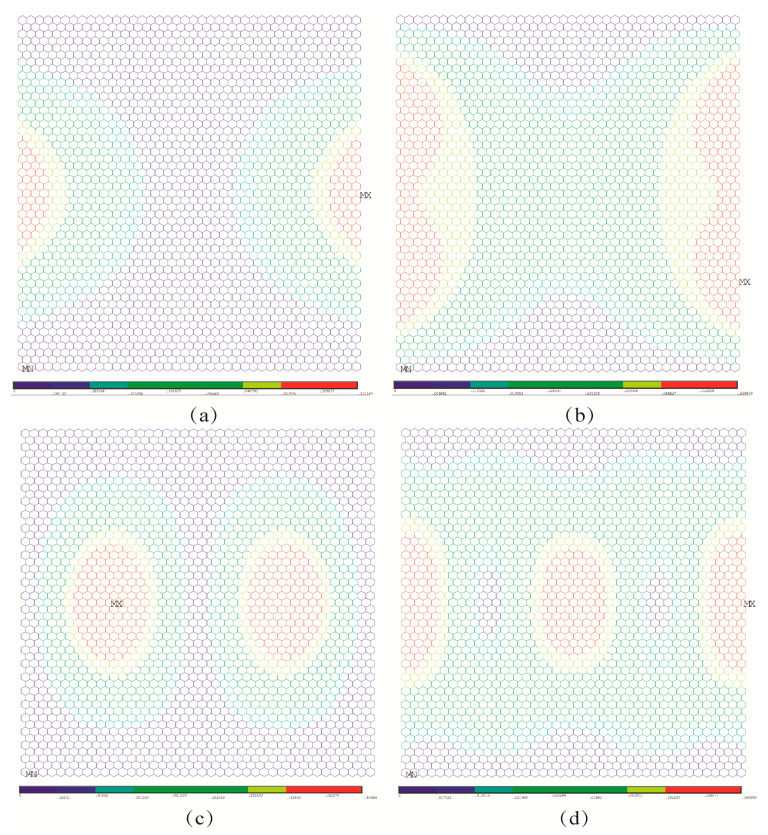

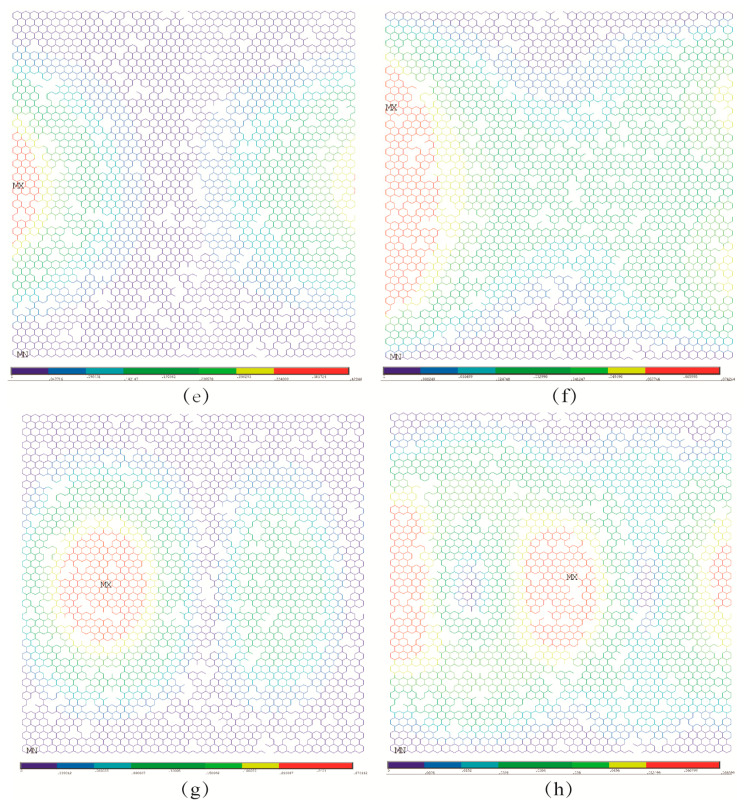
Comparison for buckling patterns under the third boundary condition ((**a**–**d**) represent the pristine graphene, while (**e**–**h**) represent the porous graphene with 5% atomic vacancy defects, respectively).

In order to compare the impacts of boundary conditions, the buckling patterns of both the porous graphene and the pristine graphene under different boundary conditions are presented in Figure 4, Figure 5 and Figure 6. As in Figure 4a, the displacement contour for the pristine graphene under the first order elastic buckling is presented, and the rotation contour is as shown in Figure 4b. The displacement and rotation contours for the pristine graphene under the fourth order elastic buckling are presented in Figure 4c,d, respectively. The parallel results for the porous graphene with 5% atomic vacancy defects are shown in Figure 4e–h. The bucking patterns of the porous graphene are similar to that of pristine graphene under the first boundary condition. In Figure 5, the differences between porous graphene and pristine graphene are not evident. The effects of the random porosities in graphene are not obvious when the boundary conditions are settled in the first and second situations.

**Figure 7:**
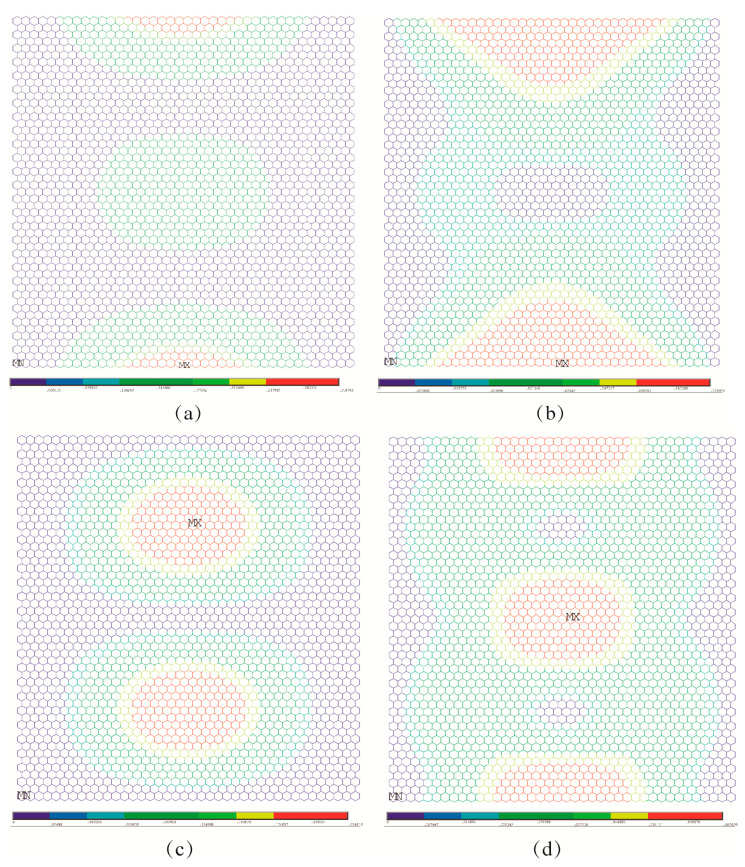

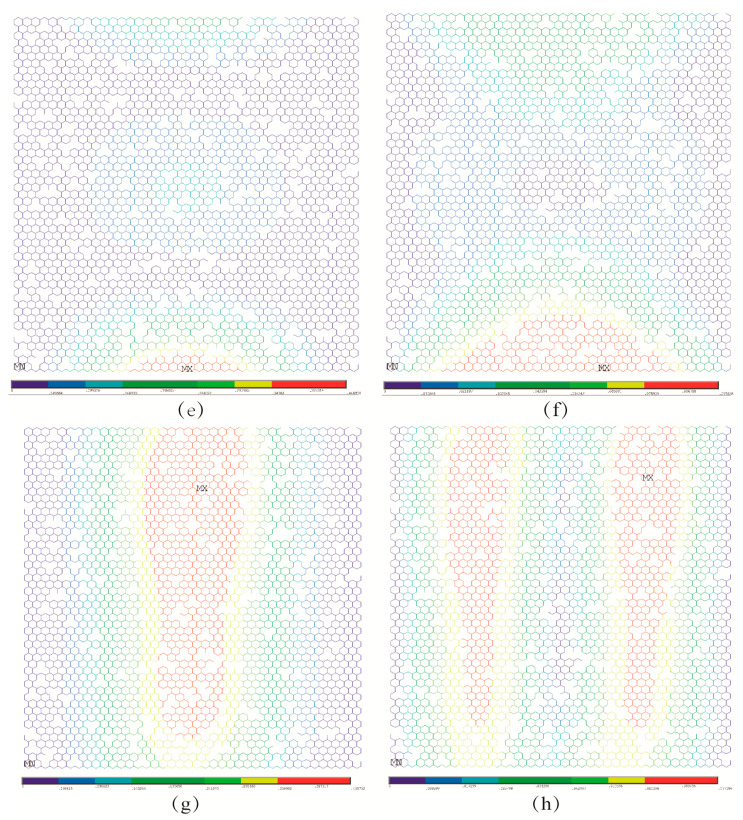
Comparison for buckling patterns under the fourth boundary condition ((**a**–**d**) represent the pristine graphene, while (**e**–**h**) represent the porous graphene with 5% atomic vacancy defects, respectively).

Under the third and fourth boundary conditions, the effects of random porosities in the buckling patterns are more apparent than those under the above-mentioned two boundary conditions. In Figure 6a–d, the displacement and rotation contour for the first and fourth order elastic buckling modes present geometrical symmetry and regularity for the pristine graphene. However, the randomly distributed atomic vacancy defects, which formed the porous graphene, play evident roles in the displacement and rotation results, as shown in Figure 6e–h. The buckling patterns are influenced by the randomly distributed atomic vacancy defects in graphene under different boundary conditions. Therefore, the comparison of buckling pattern results of pristine graphene and defective graphene is an evident index to analyze the effects of random porosities. The differences between the results in Figure 7a–h also prove the influences of random porosities in the buckling patterns. The random porosities in graphene under the third and fourth boundary conditions cause complexity and irregularity in deformation. However, the differences between porous graphene and pristine graphene are not evident in the first and second boundary conditions. On the one hand, the impacts of random porosities in the buckling patterns are sensitive to boundary conditions. The boundary condition is an efficient way to control and change the buckling patterns. On the other hand, the geometrical characteristics of graphene, such as the periodicity, symmetry, and regularity, present the capacity to defend against the influences of random porosity under the first and second boundary conditions.

The buckling patterns provide the deformation shape of porous graphene under compressive loads. For the hydrogen storage and energy harvest by the porous graphene, besides the buckling patterns, the critical buckling stress is the key factor to operate and control the process. The critical buckling stress is the compressive load at which the structure suddenly buckles or loses stability. Therefore, the critical buckling stress calculation for porous graphene needs further exploration. On the other hand, the randomly distributed porosities inevitably exist in the graphene sheets and nanoribbons with the current research and manufacturing technologies. The discrepancies caused by the numbers of random porosities are also essential to be analyzed and discussed.

### 4.2. Statistic Results

In order to further analyze the effects of random porosities in buckled graphene, the randomly distributed porosities in graphene are propagated by the repeated Monte Carlo stochastic sampling procedure. Different percentages of random porosities and boundary conditions are performed in the stochastic finite element model. The percentages of random porosities propagated in graphene are 0.2, 0.5, 1, 3 and 5%, respectively, and they are represented by P1, P2, P3, P4, and P5.

**Figure 8:**
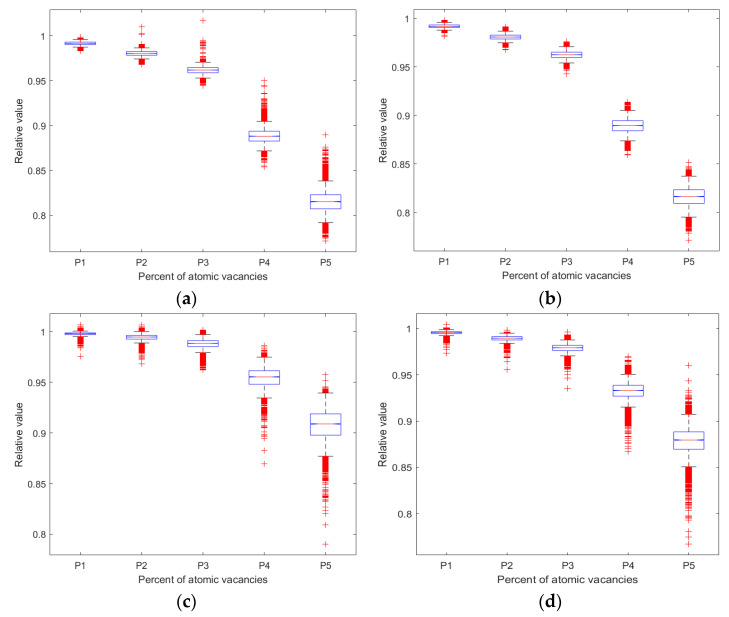
Statistic results for the relative values of critical buckling stress ((**a**–**d**) represent the first to the fourth boundary conditions B1, B2, B3, and B4; P1, P2, P3, P4, and P5 are 0.2, 0.5, 1, 3 and 5%, respectively).

In general, the atomic vacancies lead to the reduction of critical buckling stress compared with that for the pristine graphene under all four boundary conditions, since the mean of the relative value is less than one as in Figure 8. Based on the database computed by the stochastic finite element model of the graphene with randomly distributed atomic vacancy defects, the maximum, minimum, as well as the mean values, are tracked and recorded in the large sampling space. In addition, a dimensionless unit is introduced as the relative value, which computes the ratio between the results of porous graphene and pristine graphene. As the increase in the number of atomic vacancies in porous graphene, the mean of critical buckling stress in the stochastic sample space is 81.57, 81.61, 90.71 and 87.81% of those of pristine graphene for B1, B2, B3, and B4, respectively. The variances in Figure 8c,d are more evident than that in Figure 8a,b, especially when the percentage of atomic vacancies reaches 5%. Therefore, the increase in atomic vacancies causes the reduction of the mean but the amplification of variance for the relative value in the stochastic sample space.

**Figure 9:**
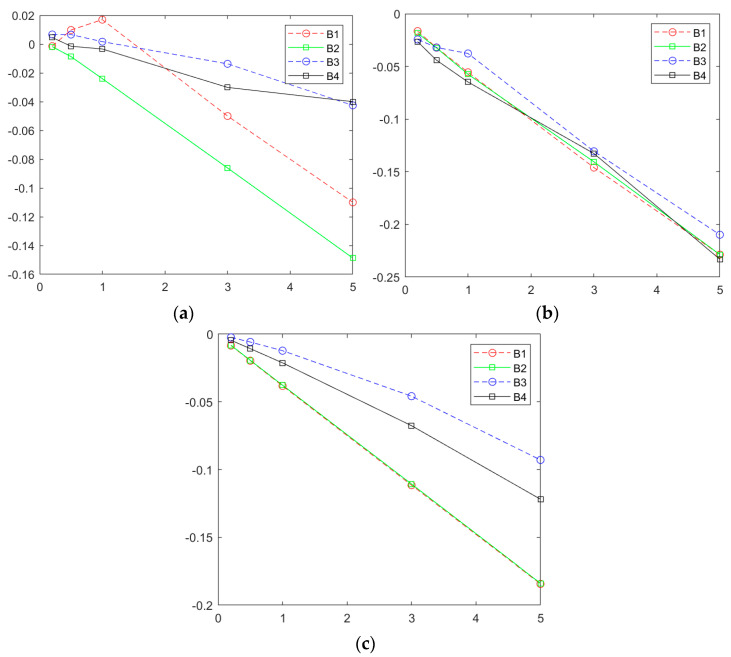
Fluctuation comparisons for the different boundary conditions ((**a**–**c**) represent the maximum, minimum, and mean values, respectively).

Furthermore, the fluctuation levels of the critical buckling stress caused by the location randomness of atomic vacancy defects are also computed by comparison with the pristine graphene. In Figure 9, both the *x* and *y* axes are dimensionless units. The *x*-axis is relevant to the percent of atomic vacancy defects; the *y* axis corresponds to the relative value between the results of porous graphene and pristine graphene. In Figure 9a, the maximum in the sample space of critical buckling stress varies more evident with the increase in the random porosities. Under the second boundary condition, the maximum in the sample space of the critical buckling stress presents a larger reduction than that under the first, third, and fourth boundary conditions. In addition, the third and fourth boundary condition is the more stable operating environment, as shown in both Figure 9a,c. Compared with the pristine graphene, both the fluctuation level of the maximum and the mean of the critical buckling stress in the sample space are lower than that under the first and the second boundary conditions.

According to the computational results of the stochastic finite element model, the specific situations caused by the location distribution of atomic vacancies are also tracked. As presented in Figure 9a, the maximum of the critical buckling stress for porous graphene presents a possibility to be larger than that of pristine graphene. Except for B2, the other three boundary conditions present strengthening effects of atomic vacancy defects in critical buckling stress. For the first boundary condition, there are 0.98% and 1.69% relative increases for porous graphene with 0.5% and 1% atomic vacancies, respectively. For the third boundary condition, 0.67, 0.66 and 0.17% increments of critical buckling stress are presented when compared with that of pristine graphene with 0.2, 0.5 and 1% atomic vacancies, respectively. The strengthening effects are also present at the fourth boundary condition. The critical buckling stress for porous graphene with 0.2% atomic vacancies increases by 0.46%.

### 4.3. Probability Density Distribution

**Figure 10:**
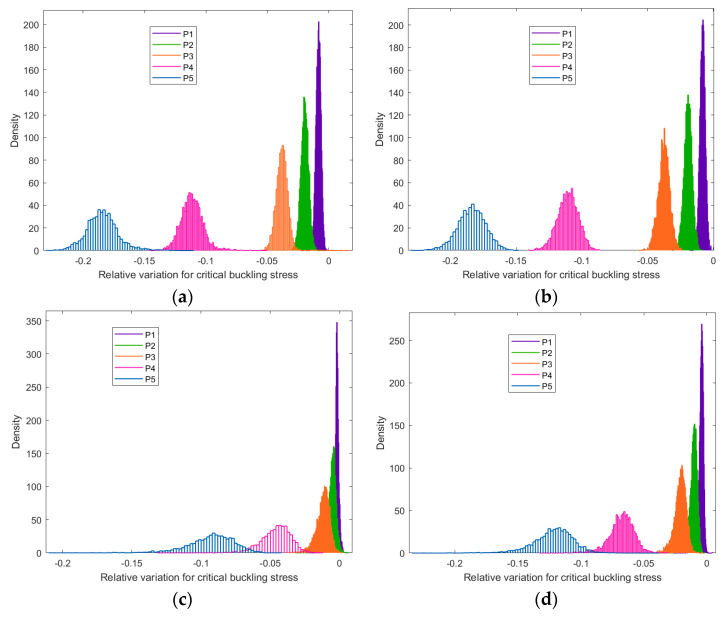
The probability density distribution of the relative variation for critical buckling stress ((**a**–**d**) represent the first to fourth boundary conditions B1, B2, B3, and B4, respectively).

In order to more precisely analyze the random porosities in the buckled graphene, the probability density distribution of the bucking critical stress under different boundary conditions is computed and presented in Figure 10. It is obvious that, when the percentage of random porosities is smaller than 1%, such as that in P1 and P2, the peak of the probability density distribution clusters nearby. However, when the percentages of random porosities are as large as that in P3, P4, and P5, the locations of the probability density distribution of critical buckling stress are settled in the identified intervals. The overlapping parts of the probability density distribution are very few in Figure 10. In other words, the amount of random porosities in graphene is the key factor to determine the interval ranges of critical buckling stress variation. In addition, the distinguishable interval ranges also prove the promising potential of critical buckling stress control through the artificial manipulation of the atomic vacancy amounts. For detailed information, please find in Appendix A.

The differences in the probability density distribution of the relative variation for critical buckling stress are also presented in Figure 10. Overall, the probability density distributions for porous graphene in Figure 10a are more approximated to those in Figure 10b, while the results in Figure 10c are more similar to those in Figure 10d. As mentioned above, the first and second boundary conditions are grouped into one set, while the third and fourth boundary conditions are grouped into another set. The relative variations for critical buckling stress in the third and fourth boundary conditions are smaller than that in the first and second boundary conditions. The discrepancies in Figure 10a,b present the chirality effects in the porous graphene. The differences in Figure 10c,d are also due to the impacts of chirality in the lattice.

In addition, the shapes of the probability density distribution in Figure 10 are approximated to the Gaussian density distribution, which well confirms the periodic and symmetrical microstructure of graphene. In addition, the results also prove the efficiency and feasibility of the stochastic sampling process of the Monte Carlo method. The symmetrical probability density distributions illustrate that the random vacancy defects are unbiasedly distributed in the entire graphene. The Monte Carlo stochastic sampling procedure provides unbiased and uniform random samples to make sure all the possible situations are taken into consideration. Furthermore, the approximated Gaussian density distribution of critical buckling stress also demonstrates that the critical buckling stress is focused on a certain peak when the percentage of the atomic vacancy defects is deterministic. Therefore, though the random porosities cause the variation in the critical buckling stress, both the interval range and peak of the probability density distribution show artificial controllability.

## 5. Conclusions

This paper proposes an efficient numerical model for the random porosity quantification and propagation in pristine graphene. Based on the huge sampling space of the stochastic finite element model, the following points can be concluded:

- The buckling patterns and critical stress of porous graphene are sensitive to boundary conditions.
- The randomly distributed atomic vacancy defects in porous graphene destroy the regularity and symmetry of buckling patterns.
- The possibility of strengthening effects in critical buckling stress is tracked under the first, third, and fourth boundary conditions.
- The distinguishable interval ranges of probability density distribution for the relative variation of the critical buckling stress prove the promising potential of artificial control by the atomic vacancy amounts.
- It is a potentially feasible method to improve the hydrogen storage and release performance by the adaptation and change in the boundary condition of the porous graphene.
- The approximated Gaussian density distribution of critical buckling stress demonstrates the stochastic sampling efficiency by the Monte Carlo method and the artificial controllability of porous graphene.
- The results of this work provide new ideas for understanding the random porosities in buckled graphene and provide a basis for energy harvest, hydrogen storage, and artificial functionalization.

## Data Availability

Data available on request from the authors.

## Appendix A

In Table A1, Table A2, Table A3 and Table A4, the additional statistical results of critical buckling stress, including the averages, maximums, minimums, and variances, are listed. The percentages of random porosities (P_1_, P_2_, P_3_, P_4_ and P_5_) propagated in graphene are 0.2, 0.5, 1, 3 and 5%, respectively. The results in Table A1, Table A2, Table A3 and Table A4 are dimensionless quantities and computed as a relative value compared with the corresponding results of pristine graphene.

**Table A1:** Statistic results for porous graphene under the first boundary condition.

	Mean	Maxi	Min	Variance
P_1_	0.9916	0.9986	0.9835	0.0704
P_2_	0.9804	1.0098	0.9678	0.1732
P_3_	0.9618	1.0169	0.9444	0.3832
P_4_	0.8886	0.9500	0.8540	0.3459
P_5_	0.8157	0.8900	0.7714	0.7096

**Table A2:** Statistic results for porous graphene under the second boundary condition.

	Mean	Maxi	Min	Variance
P_1_	0.9918	0.9982	0.9816	0.0175
P_2_	0.9807	0.9913	0.9676	0.0409
P_3_	0.9623	0.9760	0.9426	0.0824
P_4_	0.8893	0.9139	0.8594	0.2703
P_5_	0.8161	0.8513	0.7714	0.4892

**Table A3:** Statistic results for porous graphene under the third boundary condition.

	Mean	Maxi	Min	Variance
P_1_	0.9976	1.0067	0.9757	0.2198
P_2_	0.9942	1.0066	0.9680	0.5589
P_3_	0.9878	1.0017	0.9623	1.1663
P_4_	0.9542	0.9863	0.8694	5.5915
P_5_	0.9071	0.9574	0.7902	14.0503

**Table A4:** Statistic results for porous graphene under the fourth boundary condition.

	Mean	Maxi	Min	Variance
P_1_	0.9955	1.0046	0.9735	0.3041
P_2_	0.9893	0.9985	0.9559	0.7886
P_3_	0.9788	0.9965	0.9352	1.6734
P_4_	0.9324	0.9699	0.8673	7.4369
P_5_	0.8781	0.9600	0.7669	19.4867
